# Hepatic* YAP1-TFE3* Rearranged Epithelioid Hemangioendothelioma

**DOI:** 10.1155/2019/7530845

**Published:** 2019-06-23

**Authors:** Mira M. Lotfalla, Andrew L. Folpe, Karen J. Fritchie, Patricia T. Greipp, Gretchen G. Galliano, Kevin C. Halling, Taofic Mounajjed, Jorge Torres-Mora, Rondell P. Graham

**Affiliations:** ^1^Department of Laboratory Medicine and Pathology, Mayo Clinic, Rochester, MN, USA; ^2^Anatomic Pathology, Ochsner Health System, New Orleans, LA, USA

## Abstract

Epithelioid hemangioendothelioma (EHE) is an uncommon low-grade malignant vascular tumor that may arise in soft tissue/bone or visceral sites such as the liver and lung. As this tumor exhibits epithelioid morphology, it frequently causes diagnostic confusion with other epithelioid vascular neoplasms as well as carcinoma. While 90% of classic EHE are driven by a* WWTR1-CAMTA1* fusion gene, a histologically distinctive subset of EHE has been recently shown to harbor a different fusion gene,* YAP1-TFE3*. This variant is likely underrecognized given its rarity and only recent description. Notably, EHE frequently involves the liver but only one case of hepatic* YAP1-TFE3* rearranged EHE has been reported to date. We present the second case of* YAP1-TFE3* rearranged EHE affecting a 65-year-old woman and presenting as multiple liver masses, with characterization of the fusion gene at the transcriptomic and genomic levels. There are several educational points noted from this case.* YAP1-TFE3* rearranged EHE shows distinctly vasoformative foci, unlike classic EHE and mimicking angiosarcoma or epithelioid hemangioma. The tumors cells show a histiocytoid appearance with voluminous cytoplasm, similar to other* TFE3-*rearranged tumors. Finally, in the liver, this tumor may in part mimic focal nodular hyperplasia of the liver which is an underrecognized diagnostic pitfall. This report highlights the key diagnostic and genetic features of this newly recognized variant of hepatic EHE to aid pathologists in appropriately classifying these tumors.

## 1. Introduction

Epithelioid hemangioendothelioma (EHE) is an uncommon low-grade malignant vascular tumor that may arise in soft tissue/bone or visceral sites such as the liver and lung. As this tumor exhibits epithelioid morphology, it frequently causes diagnostic confusion with other epithelioid vascular neoplasms as well as carcinomas. While 90% of classic EHE are driven by a* WWTR1-CAMTA1* fusion gene, a histologically distinctive subset of EHE has been recently shown to harbor a different fusion gene,* YAP1-TFE3* [1]. This variant is likely underrecognized given its rarity and only recent description. Notably, EHE frequently involves the liver but only one case of hepatic* YAP1-TFE3* EHE has been reported to date [2]. We present an illustrative second case presenting as liver masses which shows the distinctive morphology and molecular features of this entity as well as the potential diagnostic pitfalls.

## 2. Case Presentation

**Figure 1:**
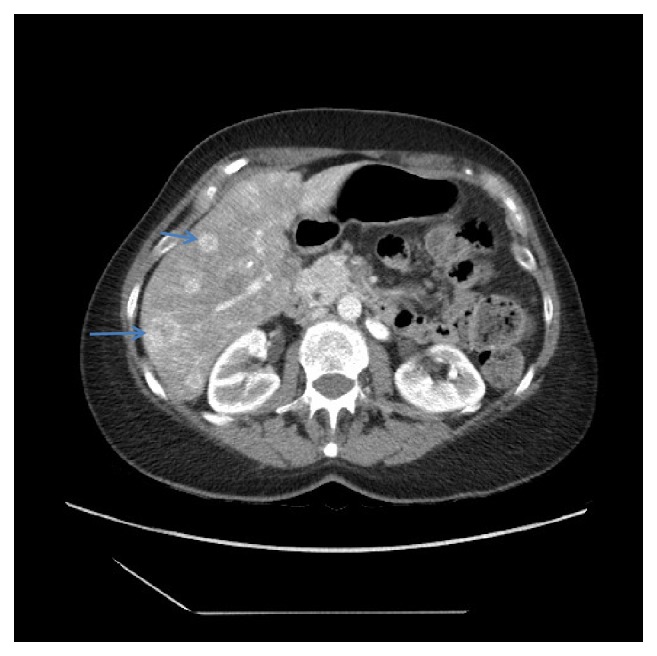
Computed tomography image showing multifocal hepatic lesions with peripheral enhancement and central hypoattenuation. Blue arrows point to calcifications.

A 65-year-old woman with multiple liver masses presented for transplantation following a diagnosis of EHE made on biopsy. Preoperative imaging revealed an enlarged liver with multifocal disease throughout the hepatic parenchyma. The lesions showed peripheral enhancement with central hypoattenuation on arterial phase and appeared isodense to the liver parenchyma on delayed phase imaging. Some of the masses showed calcifications (Figure 1). There was no significant interval change in size in the 10 months from initial diagnosis to transplantation. No chest or pelvic masses were identified elsewhere during the preoperative workup.

**Figure 2:**
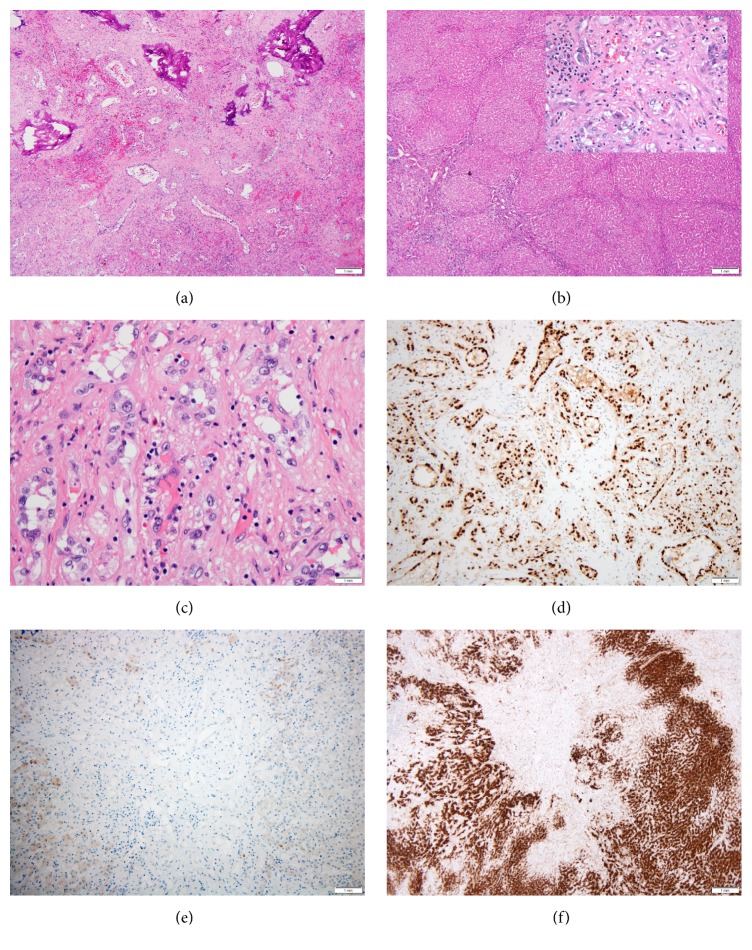
Photomicrographs of hepatic epithelioid hemangioendothelioma. (a) This mass shows one of the low magnification morphologic patterns of this tumor; well-formed vascular channels in a sclerotic matrix with focal calcification (original magnification x 100). (b) Other areas resembled focal nodular hyperplasia with a multinodular proliferation of hepatocytes around a stellate area of fibrosis (original magnification x40). (inset) The tumor cells characterized by mild nuclear atypia and abundant cytoplasm subtly infiltrate the collagen as seen in the center of this image (original magnification x400). (c) This photomicrograph shows the second phenotype of the tumor cells. Instead of well-formed vascular channels, the tumor cells are characterized by pale eosinophilic cytoplasm and intracytoplasmic vacuoles (original magnification x400). (d) The endothelial phenotype of the tumor cells is confirmed by strong and diffuse expression ERG (original magnification x100). (e) Tumor cells are negative for CAMTA1 (original magnification x100). (f) The mimic of typical focal nodular hyperplasia extended beyond hematoxylin and eosin stained stains to a map-like expression of glutamine synthetase (original magnification x40).

Pathologic examination of the liver explant disclosed multiple tan discrete nodules ranging from 0.5 to 3.0 cm in greatest dimension and involving the bilateral lobes. Low power examination revealed 2 main histologic patterns. A subset of the nodules contained well-formed vascular channels and scattered cords of tumor cells embedded in an abundantly sclerotic hyalinized matrix (Figure 2(a)), while other areas of tumor were characterized by small, irregular central hyalinized scars containing small subtle foci of neoplastic cells and a mild bile ductular reaction (Figure 2(b)). On high magnification, the tumor cells in both components exhibited mild cytologic atypia and were characterized by abundant pale cytoplasm, nuclei bearing open chromatin and visible though not large nucleoli. Scattered intracytoplasmic vacuoles were noted in the tumor cells arranged in a linear pattern (Figure 2(c)). Mitotic figures were not observed; however, focal vascular invasion by single tumor cells was noted. There was stromal lymphocytic inflammation, and focal calcification was noted in one of the lesions. The surrounding liver parenchyma had a nodular architecture, reminiscent of focal nodular hyperplasia (Figure 2(b)).

**Figure 3:**
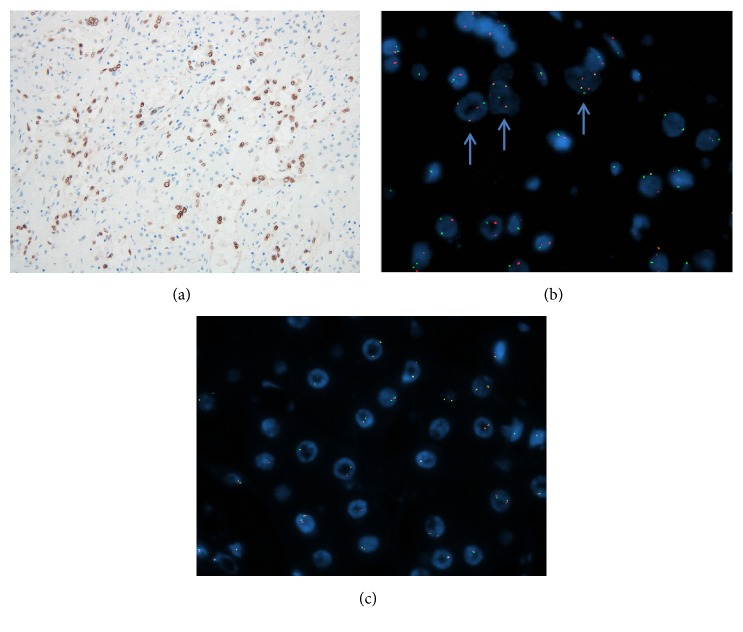
(a) Nuclear TFE3 expression by the neoplastic cells. (b) Dual fusion fluorescent in situ hybridization probes for* YAP1*- (spectrum green)* TFE3 *(spectrum orange) showing fusion of the two loci highlighted by blue arrows. (c) Split apart fluorescent in situ hybridization showing intact CAMTA1.

Immunohistochemically, the tumor cells showed an endothelial phenotype with strong expression of CD31 (clone JC70A; 1/350; Dako) and ERG (clone EPR3864; predilute; Ventana) (Figure 2(d)). The tumor nuclei were positive for TFE3 (clone MRQ-37; 1/50; Cell Marque) (Figure 3(a)), but CAMTA1 (polyclonal; 1/300; Novus Biologicals) was negative (Figure 2(e)). Glutamine synthetase (clone GS-6; 1/2000; Millipore) showed a map-like pattern of distribution in the hepatocytes surrounding small irregular areas of scar mimicking focal nodular hyperplasia (Figure 2(f)).

RNA sequencing with fusion gene analysis from formalin-fixed paraffin embedded tissue revealed an in-frame* YAP1-TFE3* fusion gene joining exon 1 of* YAP1* to exon 4 of* TFE3* and a reciprocal* TFE3-YAP1* joining exon 3 of* TFE3* to exon 2 of* YAP1*, similar to the findings of Antonescu and colleagues [1], using a published lab-developed protocol [3]. No other fusion genes, including* WWTR1-CAMTA1*, were identified. Fluorescence in situ hybridization (FISH) was performed with lab-developed split apart probes for the* TFE3* locus (bacterial artificial chromosome clones RP11-416B14, CTD-2311N12, CTD-3009K20, and CTD-2173N15; all from Abbott Molecular, IL) and dual fusion probes for* YAP1-TFE3* loci (bacterial artificial chromosome clones RP11-635D24, RP11-451F15, RP11-90M3, and RP11-750P5 along with the previously mentioned clones for* TFE3*; all from Abbott Molecular, IL) using standard techniques as previously reported by our group [4]. Interphase FISH revealed rearrangement of the* TFE3* locus in all tumor cells and* YAP1-TFE3* fusion at the genomic level (Figure 3(b)). An image of intact CAMTA1 split apart FISH performed using the same method and probes to the CAMTA1 locus is also shown (Figure 3(c)). Based on the combination of morphologic, immunophenotypic, and genetic findings, this case was classified as a* YAP1-TFE3* rearranged EHE.

## 3. Discussion

EHE, a malignant vascular neoplasm first described by Enzinger and Weiss in 1982 [5], may arise at a variety of anatomic locations including bone, soft tissue, and viscera. Although this entity is well-recognized, EHE still represents a diagnostic challenge in routine practice due to its rarity and morphologic overlap with other epithelioid tumors, including other benign and malignant epithelioid vascular neoplasms and carcinomas, especially when these tumors present at visceral sites such as the liver. The vast majority (90%) of EHE harbor the* WWTR1-CAMTA1 *fusion event [6, 7], which can be detected with molecular genetic methods or with the CAMTA1 immunostain, a reliable surrogate [8].

Recently, Antonescu and colleagues described a distinctive vascular neoplasm showing some features of EHE, but characterized by an alternate* YAP1-TFE3* fusion [1]. It is not yet entirely certain whether this unusual tumor represents a variant of EHE or an altogether unique tumor [1]. Although both tumors share epithelioid morphology and a corded growth pattern, tumors harboring the* YAP1-TFE3* fusion differ from conventional EHE inasmuch as they display overt vasoformation, consist of cells with voluminous cytoplasm, and lack the distinctive myxochondroid matrix of the latter tumor [1]. However, the natural history of conventional and* YAP1-TFE3* EHE seems to be similar, with significant potential for late distant metastasis [1].

Antonescu et al.'s seminal description of* YAP1-TFE3* EHE did not identify any tumors primary to the liver [1]. To the best of our knowledge, the only* bona fide* report of this rare tumor occurring in the liver is that of Kuo et al., who reported a morphologically typical,* TFE3*-rearranged EHE occurring in a 39-year-old woman [2]. Although another primary hepatic vascular tumor has been reported by Lee and coworkers as representing EHE showing “concomitant* YAP1-TFE3* and* WWTR1-CAMTA*1 fusions,” the morphologic and molecular genetic features of this lesion are not fully convincing, in our opinion [9].

Our case report highlights several diagnostic challenges of this variant of EHE.* YAP1-TFE3 *rearranged EHE harbors areas with well-formed blood vessels, potentially mimicking a hemangioma, an entity much more common in the liver. The biphasic appearance, however, with a second component of tumor cells arranged in cords and containing scattered intracytoplasmic lumina should trigger pathologists to consider this variant of EHE. The epithelioid morphology of the neoplastic population may also raise the possibility of other epithelioid vascular tumors such as epithelioid hemangioma, epithelioid angiosarcoma, and the conventional form of EHE with* WWTR1-CAMTA1* fusion. Although the tumor cells of epithelioid hemangioma are plump and hobnailed, they lack significant atypia. Furthermore, these tumors are uniformly vasoformative and often containing numerous eosinophils. The latter feature was not seen in this case. Additionally, epithelioid hemangioma is characterized by frequent* FOSB* gene rearrangements rather than* TFE3* fusion genes [10]. Epithelioid angiosarcoma exhibits more significant cytologic atypia and mitotic activity, including atypical mitoses. As previously mentioned, the conventional form of EHE lacks well-formed vascular channels and produces distinctive myxochondroid matrix. Finally, a combination of immunohistochemical studies including CAMTA1 and TFE3 and molecular studies assessing for* WWTR1-CAMTA1* and* YAP1-TFE3* fusions aids in the correct diagnosis.

The epithelioid morphology of EHE may also mimic involvement by primary or metastatic carcinoma. Recognizing vasoformative areas is a helpful clue to endothelial lineage, but these areas may not always be present on biopsy material. The degree of atypia in* YAP1-TFE3* rearranged EHE is usually relatively mild, and pathologists should consider performing a battery of immunostains for endothelial markers (CD31, CD34, FLI-1, and ERG) on any epithelioid neoplasm lacking keratin expression or with a uniform cytologic appearance.

Finally, low magnification examination of the explanted specimen in our patient showed some masses with a striking resemblance to focal nodular hyperplasia (FNH). This low power architecture may be deceptive, potentially resulting in a failure to recognize the underlying vascular neoplasm as the tumor cells in our case were sparsely distributed within small irregular areas of fibrous tissue and in nearby sinusoids and vessels. It has been suggested that FNH and peritumoral hyperplasia, as seen in this case, are reactive processes unified by blood flow abnormalities, and that marked angiogenesis may produce the FNH phenotype, an appealing hypothesis [11]. In this case, the absence of an abnormal artery and the presence of atypical cells percolating through the small central area of fibrous tissue and the adjacent liver parenchyma on higher magnification aided distinction from FNH. It should also be emphasized that “map-like” glutamine synthetase expression, a potential pitfall illustrated by this case, should always be interpreted in the context of other clinical and morphological findings.

*YAP1 *and* WWTR1* are orthologues and are believed to function similarly in their respective fusion genes. A review of the biology of these genes is beyond the scope of this report but* YAP1-TFE3* and* WWTR1-CAMTA1* are believed to be oncogenic drivers. In brief,* YAP1* and* WWTR1* function via TEAD transcription factors to drive expression of proproliferative and antiapoptotic genes [12].* TFE3* is one of the MiT family of transcription factors which bind to specific recognition sequences of DNA to alter gene expression. The available data indicate that, in* YAP1-TFE3*, the promoter of* YAP1* is juxtaposed to the key domains of* TFE3* to drive the development of tumors [1]. The specificity of the* YAP1*-*TFE*3 and* WWTR1-CAMTA1* fusion genes makes them useful biomarkers for diagnosis as shown in this case.

## 4. Conclusion

In conclusion, we describe a second case of* YAP1-TFE3* hepatic EHE with complete characterization of the fusion gene at the transcriptomic and genomic levels. This case also exhibited findings reminiscent of focal nodular hyperplasia, which is an underrecognized potential pitfall. Pathologists should be aware of the morphologic features of this rare and recently described variant to avoid misclassification as other vascular tumors, carcinoma and focal nodular hyperplasia. Further clinicopathologic studies are necessary to determine the behavior of this variant, especially those arising in the liver.
